# G protein signaling in tumor cell growth and metastasis

**DOI:** 10.1186/1755-8166-7-S1-I56

**Published:** 2014-01-21

**Authors:** Danny Dhanasekaran

**Affiliations:** 1 Peggy and Stephenson Cancer Center, University of Oklahoma Health Sciences Center, 975 NE 10th Street, BRC 1417, Oklahoma City, OK, 73012, USA

## 

G protein signaling has been implicated in different aspects cancer growth and progression. Our studies have identified that the G12-family of G proteins that defines the gep family of oncogenes are critically involved in tumor cell proliferation and metastasis. Defining these pathways has shown that the gep protooncogene *GNA12* is specifically involved in the proliferation of ovarian cancer cells whereas *GNA13* is involved in cancer cell metastasis. Consequently, the silencing of the *gep* proto-oncogenes potently inhibited tumor growth of ovarian cancer cells in a mouse xenograft model, thus suggesting the dominant role for the *gep* oncogenes in ovarian cancer growth and progression. In addition we demonstrate a similar role for GNA13 in the invasive migration of pancreatic cancer cells. Furthermore, we demonstrate that an eleven amino acid peptide derived from the *gep* oncogenes Gα_12/13_ can effectively disrupt LPA-stimulated oncogenic pathways. Thus, in addition to unraveling the molecular mechanism underlying cancer progression and metastasis, our results provide evidence that the G protein signaling nodes can be targeted for cancer chemotherapy.

This work was supported by grants from the National Institutes of Health (CA123233, CA 125752, CA 116984).

